# Supplementary figures and images for: Deep phosphoproteomics of Klebsiella pneumoniae reveals HipA-mediated tolerance to ciprofloxacin
Source: PLoS Pathog. 2024 Dec 12;20(12):e1012759. doi: 10.1371/journal.ppat.1012759 (PMC11717353; doi:10.1371/journal.ppat.1012759)

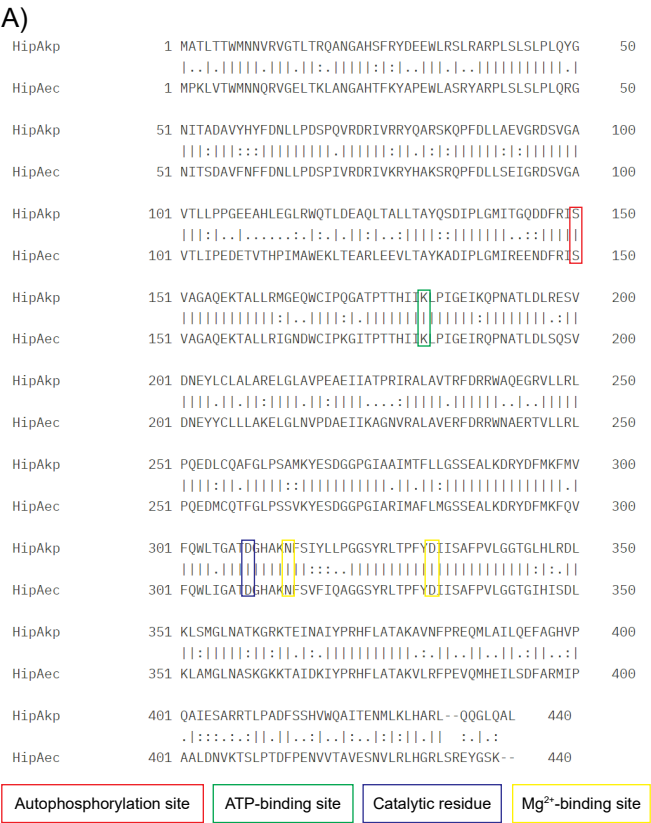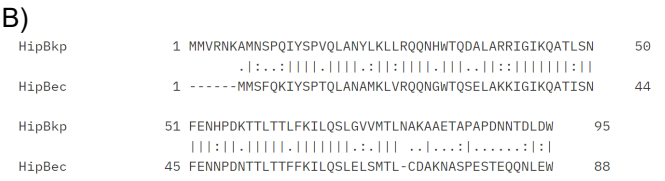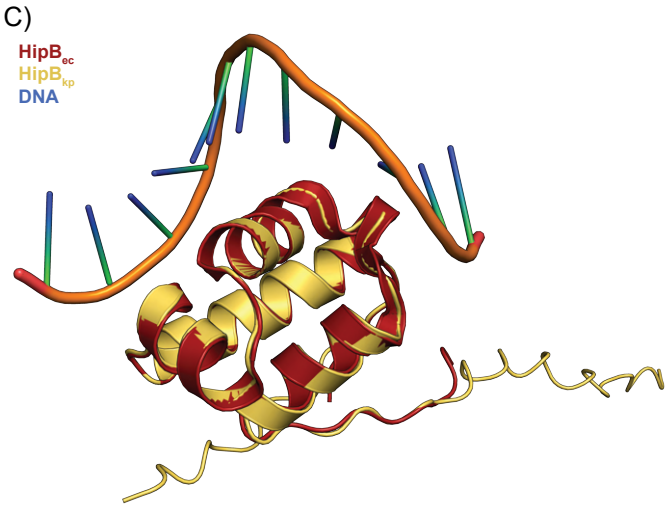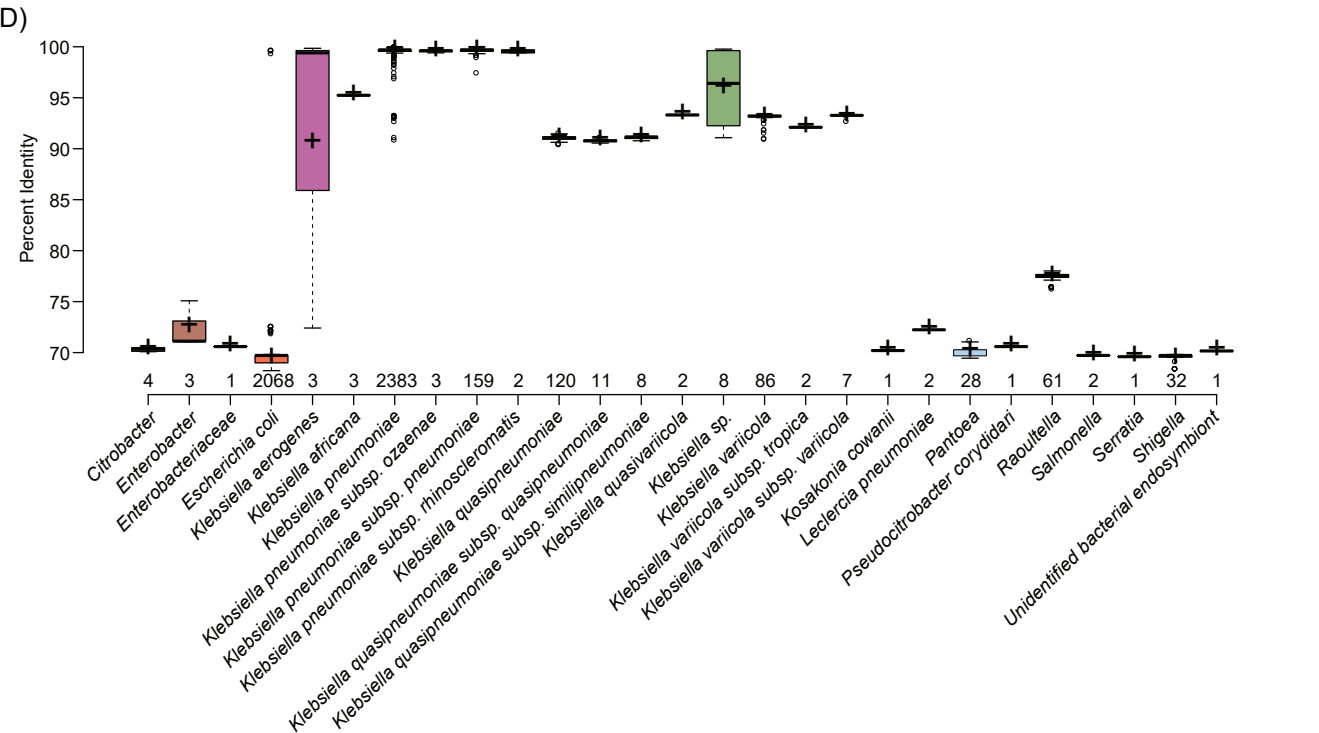

Supplement: S1 Fig — (PDF) [file ppat.1012759.s001.pdf]

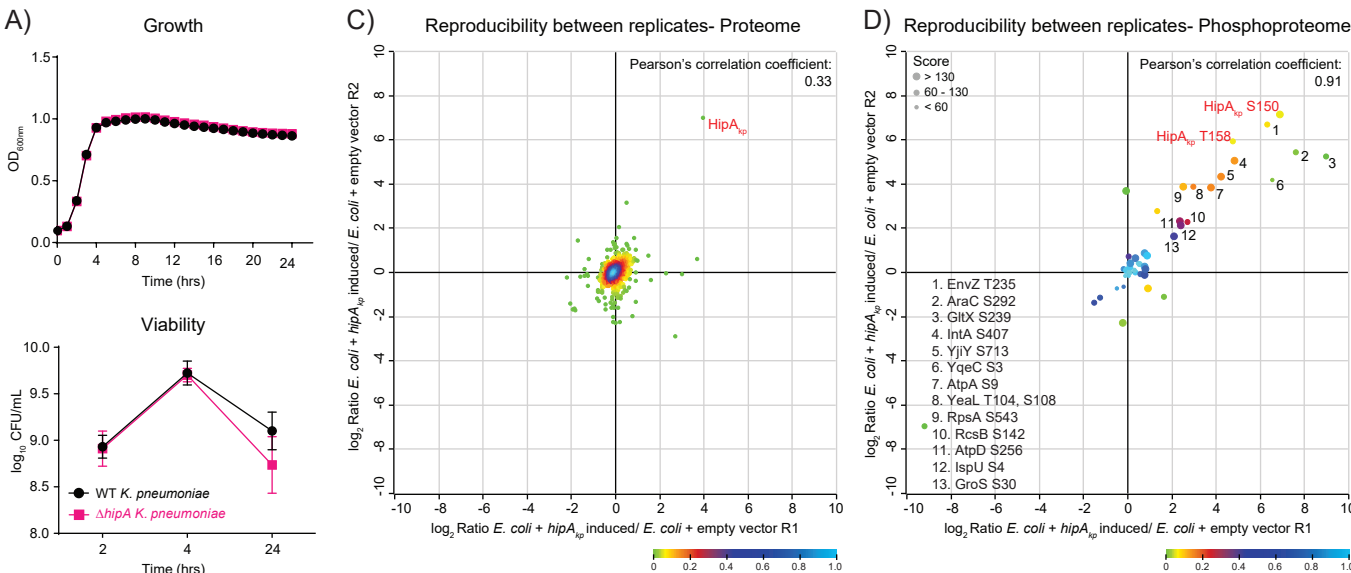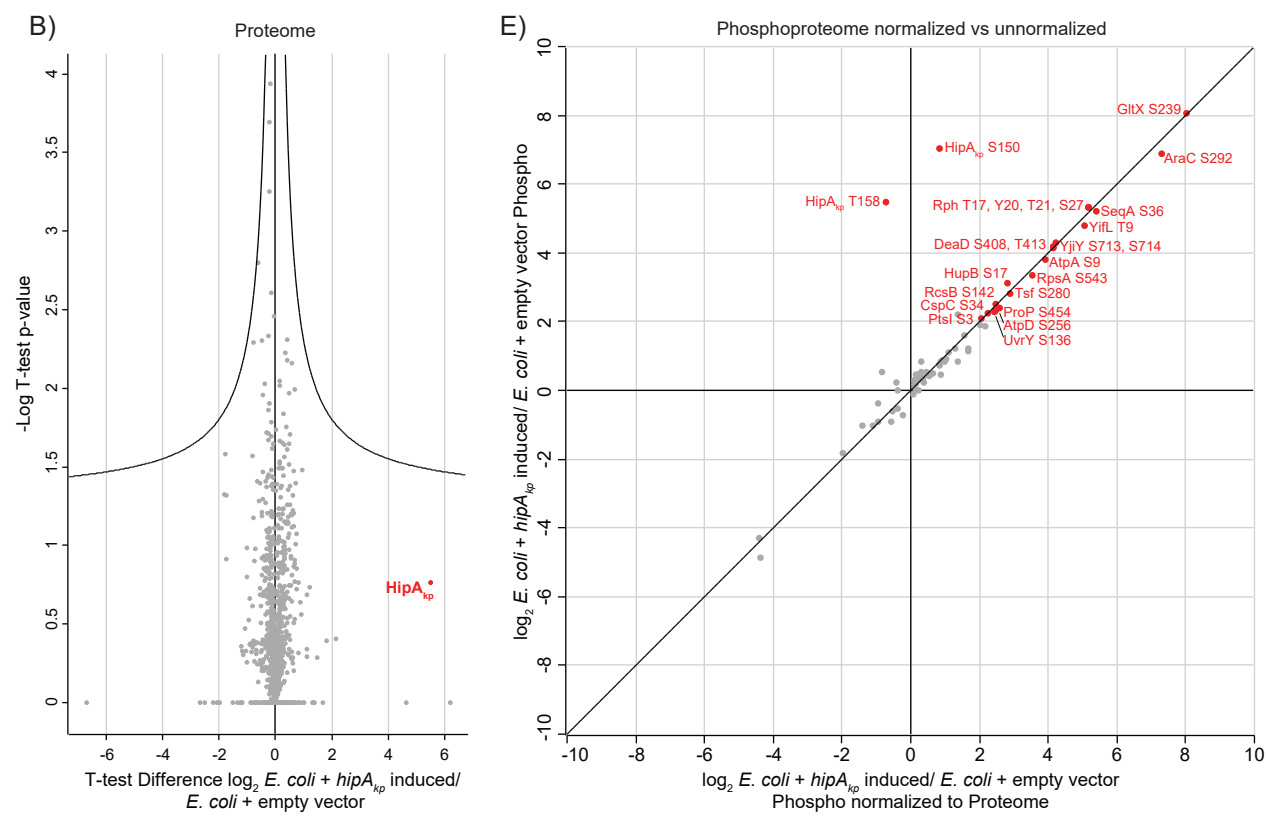

Supplement: S2 Fig — (PDF) [file ppat.1012759.s002.pdf]

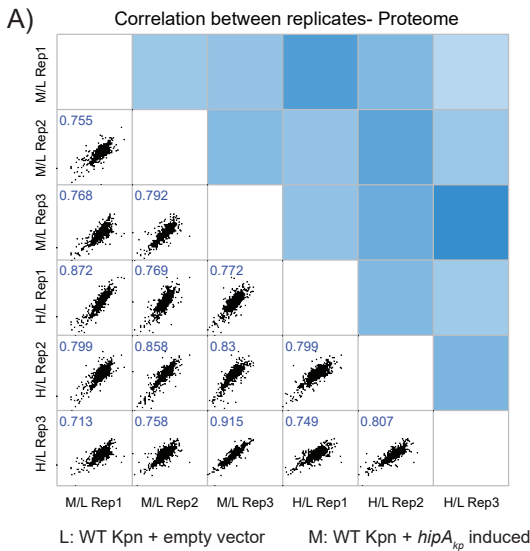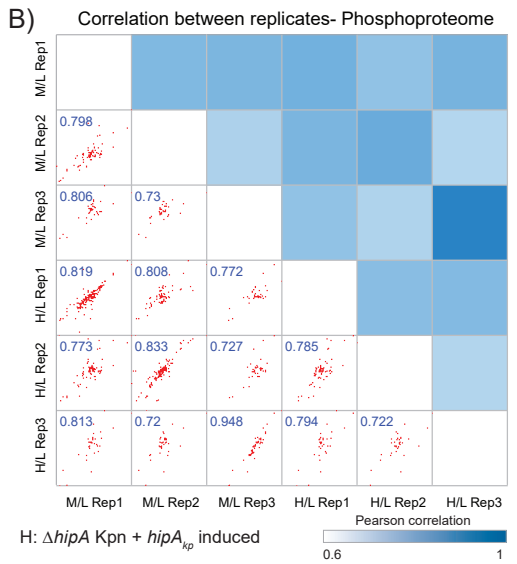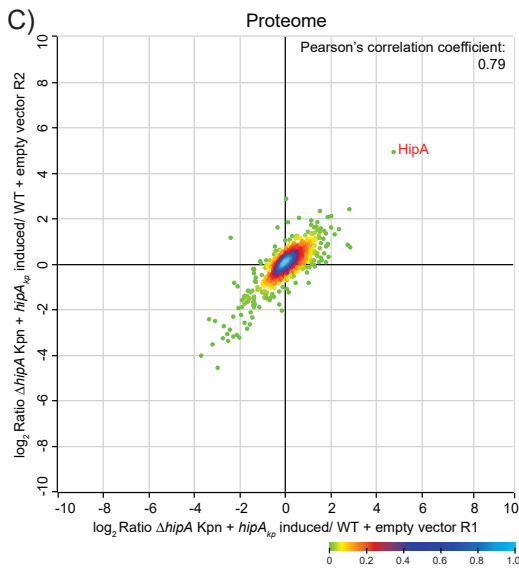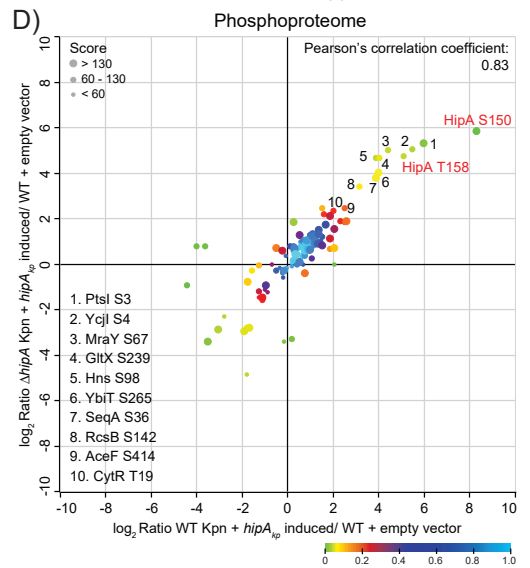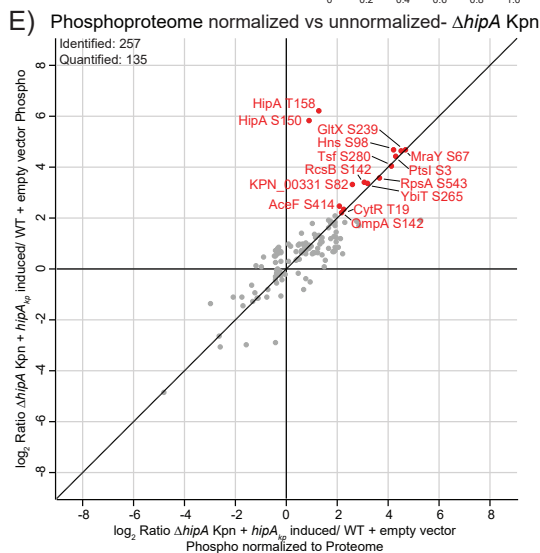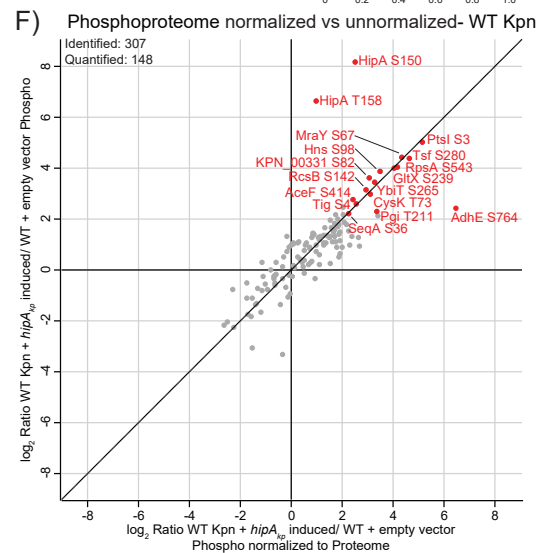

Supplement: S3 Fig — (PDF) [file ppat.1012759.s003.pdf]

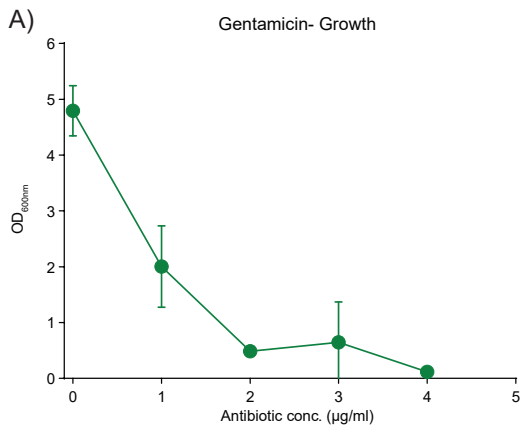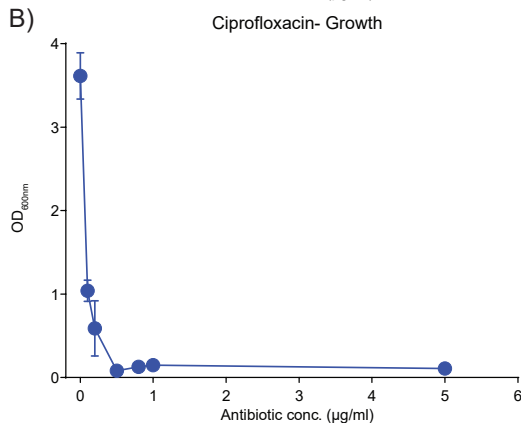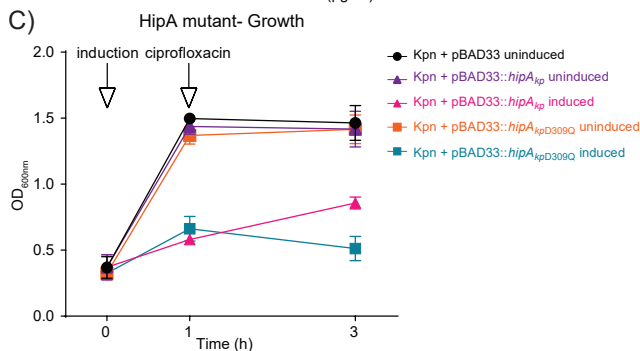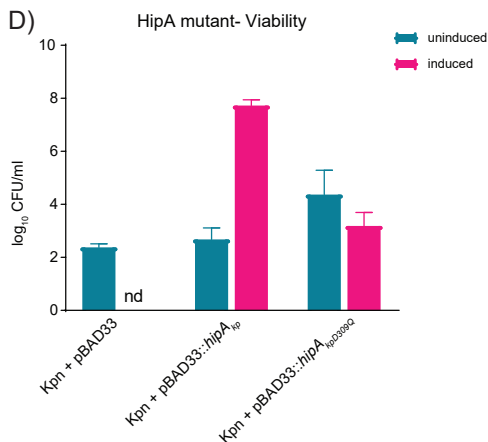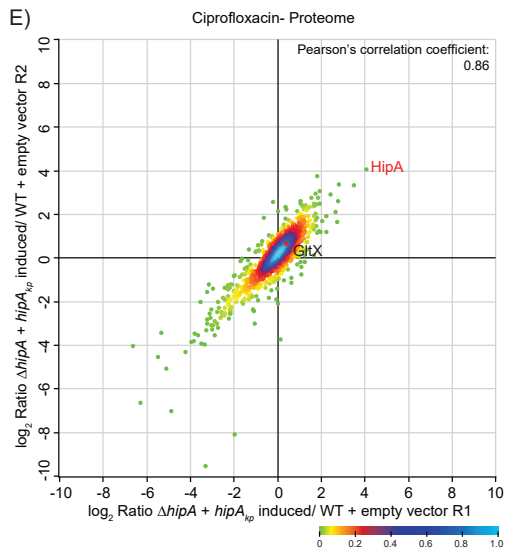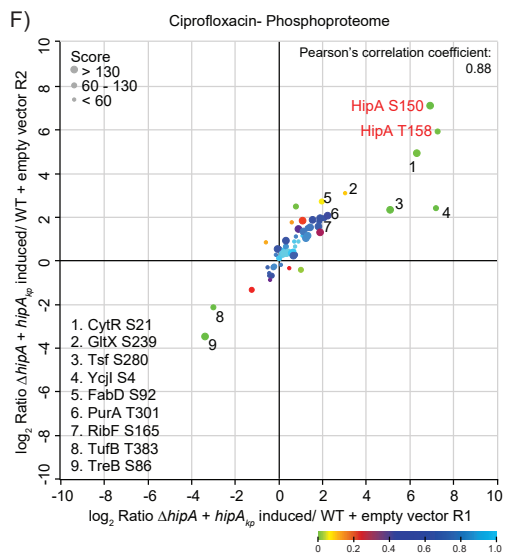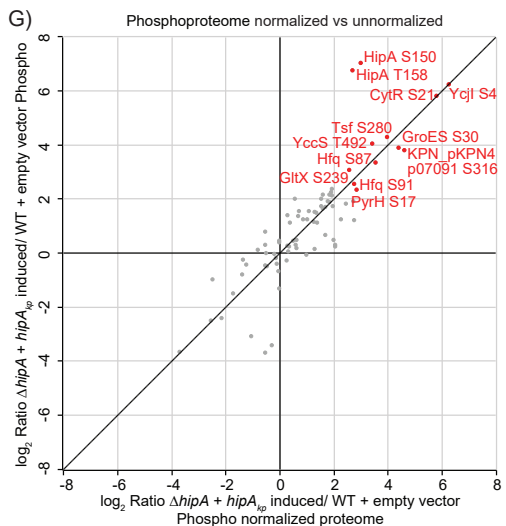

Supplement: S4 Fig — (PDF) [file ppat.1012759.s004.pdf]

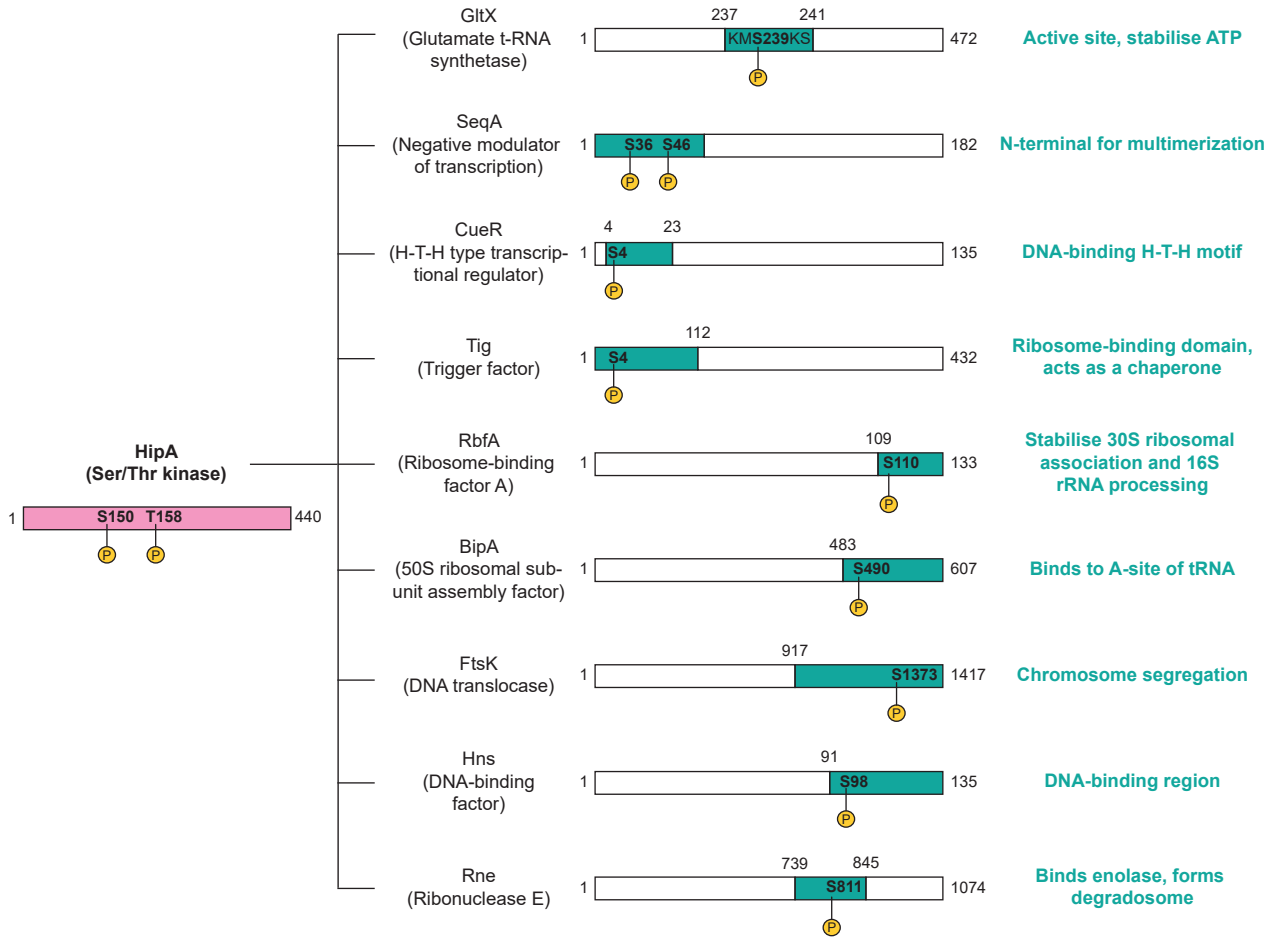

Supplement: S5 Fig — (PDF) [file ppat.1012759.s005.pdf]
