# Supplementary material for: Deep phosphoproteomics of Klebsiella pneumoniae reveals HipA-mediated tolerance to ciprofloxacin
Source: PLoS Pathog. 2024 Dec 12;20(12):e1012759. doi: 10.1371/journal.ppat.1012759 (PMC11717353; doi:10.1371/journal.ppat.1012759)
Supplement: S1 Table — (DOCX) [file ppat.1012759.s006.docx]

**S1 Table. Bacterial strains and plasmids.**

| **Strains/Plasmids** | **Genotype/ Description** | **Source/ Reference** |
| --- | --- | --- |
| **Strains** |  |  |
| *E. coli* K-12  MG1655 | Wild type- F- λ- ilvG- rfb-50 rph-1 | *E. coli* Genetic StockCenter, CGSC no. 7740 |
| *Klebsiella pneumoniae* subsp. *pneumoniae* ATCC13883 | Wild type | DSMZ |
| ATCC13883 Δ*hipA* | Unmarked in-frame deletion of *hipA* | This work |
| *E. coli* Top10 | *mcrA, Δ(mrr-hsdRMS-mcrBC), Phi80lacZ(del)M15, ΔlacX74, deoR, recA1, araD139, Δ(ara-leu)7697, galU, galK, rpsL(SmR), endA1, nupG* | Invitrogen |
| *E. coli* S17λ*pir* | *TpR SmR recA, thi, pro, hsdR-M+RP4: 2-Tc:Mu: Km Tn7 λpir* | Biomedal |
| **Plasmids** | | |
| pBAD33 | *p15A ori, f1 ori, cat promoter, araC, P_araBAD_,* CmR | (1) |
| pBAD33::*hipA_kp_* | pBAD33 P_BAD_:: *sd8 gtg hipA_kp_* | This work |
| pBAD33::*hipA_kpD309Q_* | pBAD33 P_BAD_:: *sd8 gtg hipA_kpD309Q_* | This work |
| pGOOD | pTrcHisB and pACYC184 derived plasmid, lacI, TetR | (2) |
| pGOOD::*hipB_kp_* | pGOOD P_GOOD_:: *hipB_kp_* | This work |
| pKNOCK-Km | *R6Kλ ori, oriT*, KanR | Addgene plasmid #46262(3) |
| pEXG2 | *pBR ori*, *sacB*, GmR | (4) |

**References:**

1. Guzman LM, Belin D, Carson MJ, Beckwith J. Tight regulation, modulation, and high-level expression by vectors containing the arabinose pBAD promoter. Journal of Bacteriology. 1995;177:4121-30.

2. Conte E, Landolfi G, Vincelli G, Stefan A, Hochkoeppler A. pGOODs: New plasmids for the co-expression of proteins in *Escherichia coli*. Biotechnology Letters. 2011;33:1815-21.

3. Alexeyev MF. The pKNOCK series of broad-host-range mobilizable suicide vectors for gene knockout and targeted DNA insertion into the chromosome of gram-negative bacteria. Biotechniques. 1999;26(5):824-8.

4. Rietsch A, Vallet-Gely I, Dove SL, Mekalanos JJ. ExsE, a secreted regulator of type III secretion genes in *Pseudomonas aeruginosa*. Proceedings of the National Academy of Sciences. 2005;102(22):8006-11.
