# Supplementary material for: Deep phosphoproteomics of Klebsiella pneumoniae reveals HipA-mediated tolerance to ciprofloxacin
Source: PLoS Pathog. 2024 Dec 12;20(12):e1012759. doi: 10.1371/journal.ppat.1012759 (PMC11717353; doi:10.1371/journal.ppat.1012759)
Supplement: S2 Table — (DOCX) [file ppat.1012759.s007.docx]

**S2 Table. DNA oligonucleotides.**

| **Name** | **Sequence 5’-3’** | **Primer description** |
| --- | --- | --- |
| HipA.kpn.SD8.GTG.  pBAD33-for | tcggtacccggggatccta*AAATAAGGAGGAAAAAAAAA*GTGGCGACGCTTACCACC | For amplification of *hipA_kp_* and cloning into pBAD33; the shine dalgarno sequence is in Italics, underlined is the substituted nucleotide |
| HipA.kpn.pBAD33-rev | catgcctgcaggtcgactCTATAGCGCCTGCAGCCC | For amplification of *hipA_kp_* and cloning into pBAD33 |
| hipBkp.pGOOD-for | cccatatggtaccagctgcaTTACCAGTCAAGGTCCGTGT | For amplification of *hipB_kp_* and cloning into pGOOD |
| hipBkp.pGOOD-rev | cgataaggatccgagctcgaATGAATTCCCCGCAGATATACAGT | For amplification of *hipB_kp_* and cloning into pGOOD |
| pBAD F | CGCAACTCTCTACTGTTTCTC | Sequencing primer for pBAD33 |
| pBAD R | CCGCTTCTGCGTTCTG | Sequencing primer for pBAD33 |
| pGOOD.seq-for | GCCGCCAGGCAAATTCTGT | Sequencing primer for pGOOD |
| pGOOD.seq-rev | TGGGGGGTTCTCATCATCATC | Sequencing primer for pGOOD |
| KpnATCC13883_hipA ko XbaI IF F | tggcggccgctctagGGCGGCAGAGGACCATTTCCA | For amplifying *hipA* flanking regions and cloning into pKNOCK-Km |
| KpnATCC13883_hipA ko overlap F | aactcgagccgcaagcatgctgaaCCAGGTGGTAAGCGTCGCCAT | For amplifying *hipA* flanking regions and cloning into pKNOCK-Km |
| KpnATCC13883_hipA ko overlap R | ttcagcatgcttgcggctcgagttCAAGGGCTGCAGGCGCTATAG | For amplifying *hipA* flanking regions and cloning into pKNOCK-Km |
| KpnATCC13883_hipA ko XbaI IF R | atccactagttctagTATGCCTCGAAGGGCGACTGG | For amplifying *hipA* flanking regions and cloning into pKNOCK-Km |
| KpnATCC13883_hipA inside F | AGCAGATGCTGGCGATCCTC | For verification of the deletion of *hipA* from the chromosome |
| KpnATCC13883_hipA ko outside R | GAACTACCCGATTGACGGCG | For verification of the deletion of *hipA* from the chromosome |
| pBAD33 hipAkp D309Q F | GGGGGCGACGcagGGTCACGCAAA | For mutant *hipA* D309Q  Underlined: mutated codon |
| pBAD33 hipAkp D309Q R | GTCAGCCACTGGAAGACCATGAACTTCA | For mutant *hipA* D309Q |

The sequence part of the gene is shown in uppercase. The sequence part of the vector is indicated in lowercase.
