# Supplementary material for: Deep phosphoproteomics of Klebsiella pneumoniae reveals HipA-mediated tolerance to ciprofloxacin
Source: PLoS Pathog. 2024 Dec 12;20(12):e1012759. doi: 10.1371/journal.ppat.1012759 (PMC11717353; doi:10.1371/journal.ppat.1012759)
Supplement: S3 Table — (DOCX) [file ppat.1012759.s008.docx]

**S3 Table. Overview of experiments for LC-MS/MS measurements of proteome and phosphoproteome analysis.**

| **Exp. No.** | **Experiment name** | **Data to Figure** | **No. of replicates** | **Type of Samples and Dimethyl Labeling^a^** |  |
| --- | --- | --- | --- | --- | --- |
| **Overexpression of *hipA_kp_* in *E. coli*** | | | | | |
| **1.1** | HipA_kp_ o/e in *E. coli* | fig. S2 | 4  (2- LysC  2- Chymotrypsin) | L: WT *E. coli* + empty vector  H: WT *E. coli* + pBAD33::*hipA_kp_* induced |  |
| **Overexpression of *hipA_kp_* in *K. pneumoniae*** | | | | | |
| **2.1** | HipA_kp_ o/e in *K. pneumoniae* | Fig. 3, 5, fig. S3 | 6  (3- Trypsin  3- Chymotrypsin) | L: WT Kpn + empty vector  M: WT Kpn + pBAD33::*hipA_kp_* induced  H: ∆*hipA* Kpn + pBAD33::*hipA_kp_* induced |  |
| **2.2** | HipA_kp_ o/e and Ciprofloxacin treatment in *K. pneumoniae* | Fig. 4, 5, fig. S4 | 4  (2- Trypsin  2- Chymotrypsin) | L: WT Kpn + empty vector  M: ∆*hipA* Kpn + pBAD33::*hipA_kp_* uninduced  H: ∆*hipA* Kpn + pBAD33::*hipA_kp_* induced |  |
| **2.3** | HipA_kp_ o/e in *K. pneumoniae* | Fig. 5 | 4  (2- Trypsin  2- Chymotrypsin) | L: WT Kpn  M: WT Kpn + pBAD33::*hipA_kp_* uninduced  H: WT Kpn + pBAD33::*hipA_kp_* induced |  |

^a^ L: Light, M: Medium, H: Heavy label.
