# Supplementary material for: Deep phosphoproteomics of Klebsiella pneumoniae reveals HipA-mediated tolerance to ciprofloxacin
Source: PLoS Pathog. 2024 Dec 12;20(12):e1012759. doi: 10.1371/journal.ppat.1012759 (PMC11717353; doi:10.1371/journal.ppat.1012759)
Supplement: S4 Table — (DOCX) [file ppat.1012759.s009.docx]

**S4 Table. Analysis of proteome and phosphoproteome data from *Klebsiella pneumoniae*.**

| **Experiment No.** | **2.1** | **2.2** | **2.3** |
| --- | --- | --- | --- |
| **Identified P-sites** | | | |
| **Total p-sites** | 747 | 547 | 686 |
| **p-sites (PEP>0)** | 738 | 542 | 670 |
| **p-sites (Class I =>0.75)** | 517 | 393 | 470 |
| **Identified proteins** | | | |
| **Total proteins** | 1,755 | 1,889 | 1,868 |
| **Phosphoproteins** | 417 | 325 | 400 |
