# Supplementary material for: Deep phosphoproteomics of Klebsiella pneumoniae reveals HipA-mediated tolerance to ciprofloxacin
Source: PLoS Pathog. 2024 Dec 12;20(12):e1012759. doi: 10.1371/journal.ppat.1012759 (PMC11717353; doi:10.1371/journal.ppat.1012759)
Supplement: S5 Table — (DOCX) [file ppat.1012759.s010.docx]

**S5 Table. List of putative substrates of HipA_kp_ in *K. pneumoniae.***

| **UniProt protein ID** | **Protein + P- amino acid** | **Protein name** | **Occurrence*** |
| --- | --- | --- | --- |
| A6TC43 | GltX S239 | Glutamate--tRNA ligase | 10 |
| A6T928 | HipA S150, S158 | Persistence to inhibition of murein or DNA biosynthesis, DNA-binding regulator | 10, 9 |
| A6TBT4 | RcsB S142 | Transcriptional regulatory protein RcsB | 10 |
| A6T4X2 | Tsf S280, S283 | Elongation factor Ts | 10, 7 |
| A6T6R6 | YbiT S265 | Putative ATP-binding component of a transport system | 10 |
| A6T820 | YcjI S4 | Murein peptide amidase A | 10 |
| A6T5I3 | HupB S17 | DNA-binding protein HU-beta, NS1 (HU-1) | 9 |
| A6T4N0 | MraY S67 | Phospho-N-acetylmuramoyl-pentapeptide-transferase | 9 |
| A6T703 | RpsA S543 | 30S ribosomal protein S1 | 9 |
| A6T4Q8 | AceF S414 | Acetyltransferase component of pyruvate dehydrogenase complex | 8 |
| A6T5P5 | CueR S4 | HTH-type transcriptional regulator CueR | 8 |
| A6TES8 | Fis S14 | DNA-binding protein Fis | 8 |
| A6TG70 | HemN S454 | Coproporphyrinogen-III oxidase | 8 |
| A6T8X0 | LacZ S4 | Beta-galactosidase 1 | 8 |
| A6TEI6 | RbfA S110 | Ribosome-binding factor A | 8 |
| A6T5H9 | Tig S4 | Trigger factor | 8 |
| A6TGC6 | RpmE S65 | 50S ribosomal protein L31 | 7 |
| A6T6D2 | SeqA S36, S46 | Negative modulator of initiation of replication | 7, 5 |
| A6TAK6 | AdhE S764 | Aldehyde-alcohol dehydrogenase | 6 |
| A6TG38 | AtpA S9 | ATP synthase subunit alpha | 6 |
| A6T685 | CspE S34 | Cold shock protein E | 6 |
| A6TGC4 | CytR S21 | Regulator for deo operon, udp, cdd, tsx, nupC, and nupG | 6 |
| A6T6Y7 | FtsK S1373 | DNA translocase FtsK | 6 |
| A6T6Y6 | Lrp S30 | Regulator for leucine (Or lrp) regulon and high-affinity branched-chain amino acid transport system | 6 |
| A6TC52 | PtsI S3 | Phosphoenolpyruvate-protein phosphotransferase | 6 |
| A6T9X6 | RnfC S494 | Ion-translocating oxidoreductase complex subunit C | 6 |
| A6T688 | TatE S65 | Probable Sec-independent protein translocase protein TatE | 6 |
| A6T8N9 | YdaA S277 | Conserved protein, adenine nucleotide-binding domain | 6 |
| A6TCJ3 | YfiF S106 | Putative tRNA/rRNA methyltransferase | 6 |
| A6TG74 | BipA S490 | 50S ribosomal subunit assembly factor BipA | 5 |
| A6T533 | FrsA S7 | Esterase FrsA | 5 |
| A6TAK8 | Hns S98 | DNA-binding protein | 5 |
| A6T7E5 | Rne S811 | Ribonuclease E | 5 |
| A6TGW7 | Ssb S145 | Single-stranded DNA-binding protein | 5 |

*Detected in different replicates of phosphoproteome datasets of experiment 2.1-2.3, in total 10.
