# Supplementary material for: Deep phosphoproteomics of Klebsiella pneumoniae reveals HipA-mediated tolerance to ciprofloxacin
Source: PLoS Pathog. 2024 Dec 12;20(12):e1012759. doi: 10.1371/journal.ppat.1012759 (PMC11717353; doi:10.1371/journal.ppat.1012759)
Supplement: Attachment — Submitted filename: Response to Reviewers Comments FINAL.pdf [file ppat.1012759.s012.pdf]

PPATHOGENS-D-24-01163: Response to Reviewers' Comments

Our text is marked in red color.

Editor's Comments
Thank you very much for submitting your manuscript "Deep phosphoproteomics of Klebsiella pneumoniae reveals HipA-mediated tolerance to ciprofloxacin" for consideration at PLOS Pathogens. Your manuscript was reviewed by members of the editorial board and by four independent reviewers. In light of the reviews (below), we would like to invite the resubmission of a substantially revised version that takes into account the reviewers' comments.

**Please address all the concerns of the reviewers; specifically, you must provide missing information on the statistical analyses as described by Reviewer 4, assay the function of HipA with a kinase mutant as noted by Reviewers 2 and 3, and examine HipA production in antibiotic resistant strains also as mentioned by Reviewers 2 and 3.**

We thank the editors for giving us the opportunity for revision and the reviewers for their time to carefully read the manuscript and provide their constructive and thoughtful comments and recommendations, which we appreciate. We believe that we addressed all of the reviewer's suggestions and concerns. Substantial changes were made to the manuscript, which we believe significantly improved its clarity and quality. These changes include but are not limited to additional experiments to analyze a kinase mutant and HipA levels (Reviewers 2 and 3), the missing information on the statistical analysis and a re-analysis of the normalized data as requested by Reviewers 3 and 4. Please find our point-to-point response below.

**Part I - Summary**

Reviewer #1: Very nice work. I suggest "accept" upon the satisfaction of several minor suggestions.

We appreciate the very positive assessment of our work by Reviewer #1.

Reviewer #2: In this study, Nashier et al. show that functional conservation of the HipA/B operon in Klebsiella pneumoniae. By overexpressing HipA, they denmonstrate that HipA overexpression is toxic, and the toxicity of HipA overexpression is rescued by the overexpression of HipA antitoxin HipB. Notably, the overexpression of HipA led to tolerance to the antibiotic ciprofloxacin, which is commonly used for the treatment of Klebsiella pneumoniae. To illuminate the role of HipA in antibiotic tolerance, the authors used quantitative mass spectrometry to comprehensively identify changes in proteome and phosphoproteome. These analyses confirmed the kinase activity of HipA, and importantly, identification of many dozens of HipA substrates. These high-confidence HipA substrates are involved in diverse biological pathways, implying a role of HipA in their regulation.
Understanding the mechanisms of antibiotic resistance is of major importance, and this work provides a major resource for understanding the role of HipA-catalyzed phosphorylation in antibiotic resistance. Technically, mass spectrometry analyses are performed at impressive depth and the results are highly reproducible. I believe that work will be of broad interest to researchers across fields.

We thank the Reviewer #2 for the positive evaluation of our study and for recognizing the depth and reproducibility of the mass spectrometry.

Reviewer #3: The manuscript „Deep phosphoproteomics of Klebsiella pneumoniae reveals HipA-mediated tolerance to ciprofloxacin" by Nashier et al. presents the results of the proteomic and phosphoproteomic study of the HipA protein in Klebsiella pneumoniae, which by homology to the E.coli was predicted to be a kinase. The authors show that HipA overexpression indeed leads to phosphorylation changes, and the datasets comparing bacteria overexpressing HipA to wild type and the HipA overexpressors exposed to antibiotics to those that weren't provide a valuable resource for further studies. The presented data are solid but there are a few issues that should be resolved before publication.

We thank the Reviewer #3 for the favorable evaluation of the work and the solidity of the data.

Reviewer #4: The study investigates phosphorylation of HipA-associated proteins in the globally important bacterial pathogen, Klebsiella pneumoniae. The study is well presented and well written; however, the text switches between experiments with Klebsiella and E. coli, which is challenging for the reader. The study is highly descriptive for the phosphorylation studies, it would be nice to see one step further by disruption of a phospho site and the impact. Additionally, key information on the number of replicates for total proteome and phosphoproteome analysis is needed, as well as clarification and robustness in data analysis for normalization, false discovery rate correction, and phosphosite localization probability do not appear to be included. These analyses could drastically change the findings of the paper.

We thank the Reviewer #4 for a positive assessment of the study and suggestions for clarification of statistics.

**Part II – Major Issues: Key Experiments Required for Acceptance**

Reviewer #1: NA

Reviewer #2: I have a few comments that should be addressed before the manuscript is accepted for publication.

1. To directly demonstrate a role of HipA kinase activity in Klebsiella pneumoniae, it would be useful to show that kinase dead mutant of HipA is not toxic to Klebsiella pneumoniae, and unable to provide resistance to ciprofloxacin.

To address the reviewer's comment, we have performed site directed mutagenesis of pBAD33::$hipA_{kp}$ to introduce a substitution of the catalytic residue 309 (D309Q), sequence confirmed and cloned it into *K. pneumoniae*. Three independent experiments revealed a phenotypic difference between the mutant and wild-type *hipA* allele; in addition, HipAD309Q did not provide the level of ciprofloxacin tolerance as observed for wild-type HipA (Supplementary figures S4C and S4D). We note that the contribution of the conserved residues of $HipA_{kp}$ involved in ATP-binding, $Mg^{2+}$-binding, catalytic activity, and the autophosphorylation site to toxicity of HipA remains to be determined in future studies. The degree of the phenotype might be more pronounced when introducing more mutations.

2. Perhaps I missed it, but it is unclear to me whether there is any data about the expression of HipA/B in Klebsiella pneumoniae isolated from antibiotic-sensitive and anti-biotic resistance patients?

We thank the Reviewer for this interesting thought. We conducted a comprehensive review of the literature but did not find any publications that address the analysis of *hipAB* expression in *K. pneumoniae* isolated from patients who either respond or do not respond to antibiotic treatment. It is important to mention that obtaining such data is presumably very challenging, as persister cells are present in extremely low numbers under specific conditions (e.g. prolonged antibiotic treatment) in vitro and likely within the patient.

Reviewer #3: Detailed comments:

1. General: the increase in phosphorylation of the proteome when HipA is overexpressed is not a direct proof that it is, in fact, a kinase. There is a lot of indirect evidence pointing to that but this shortcoming should be discussed by the authors.

To address the Reviewer's suggestion, we have now explained in the Discussion that additional experiments are required for a more detailed analysis of $HipA_{kp}$ activity (lines 380-383). In addition to phosphoproteomics data that clearly show upregulation of multiple phosphorylation sites upon kinase overproduction, the revised manuscript contains data showing that a mutation of the $HipA_{kp}$ residue 309, which has been demonstrated to be critical for HipA kinase activity in *E. coli,* has a phenotypic effect and reduces tolerance against ciprofloxacin (Supplementary figures S4C and S4D). Furthermore, since we cannot exclude action of another kinase, we recognize that it is important to refer to upregulated phosphorylation sites as "putative" or "potential" HipA substrates, which we do throughout the manuscript.

2. The authors note the slower growth and lower viability in K. pneumoniae overexpressing HipA, and attribute it to the toxicity of HipA. The same (slow growth) was observed in E. coli, is that also because HipA from K. pneumoniae is toxic to E. coli, or overexpression of E. coli HipA in E. coli would have the same effect?

The Reviewer is correct: $HipA_{kp}$ from *K. pneumoniae* is also toxic when overexpressed in *E. coli*. Previous reports have shown that overproduced $HipA_{ec}$ is toxic to *E. coli* (PMID- 17041039, 30206139).

3. Overexpression of HipA results in HipA phosphorylation on S150 and T158 both in E.coli and K. pneumoniae. Are the phosphopeptides unique to K. pneumoniae HipA? Does it mean that HipA is constitutively autophosphorylated? Can these phosphosites be detected in WT (which, after all, expresses HipA) using targeted proteomics?

The HipA phosphopeptide (RISVAGAQEKTALL) that carries residues S150 and T158 is identical between *E. coli* and *K. pneumoniae* and is therefore not unique. Both phosphosites were also detected in the K. pneumoniae WT. Please find below a graph we prepared to illustrate the intensities of phosphorylated and unphosphorylated peptides in WT ("L" channel) and *$hipA_{kp}$* overexpression data ("M" and "H" channels) (**Rebuttal letter Figure 1**). One could argue that this phosphorylation is constitutive; however, we note that the intensity of this phosphopeptide is 100-1000x higher upon overexpression of $HipA_{kp}$ ("M" and "H" channels). This means that the occupancy of these sites in the "L" channel is negligible and we would therefore not use the term "constitutive" phosphorylation.

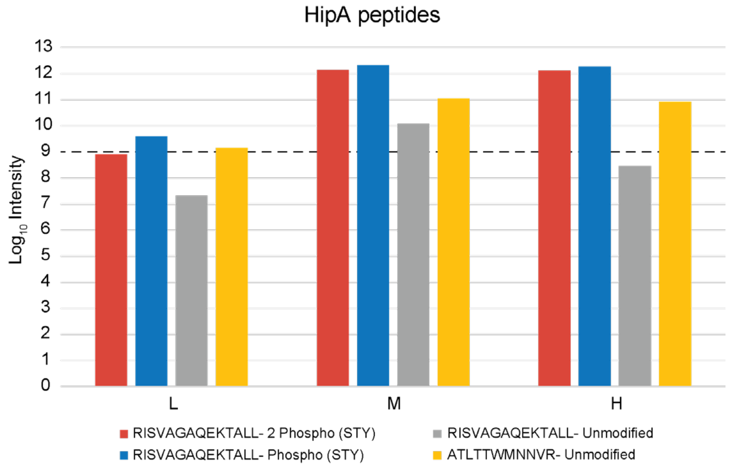


**Rebuttal letter Figure 1.** $Log_{10}$ intensity values of HipA peptides derived from L: WT + empty vector, M: WT + $HipA_{kp}$ o/e and H: Δ*hipA* + $HipA_{kp}$ o/e samples

4. If HipA overexpression leads to ciprofloxacin resistance, is the reverse also true (is HipA overexpressed in the resistant strains)?

We thank the Reviewer for raising this interesting question, which appears to have been virtually not addressed for HipA proteins in the literature. We found one study that reports increased *hipAB*$_{kp}$ expression levels after 1 h of gentamicin treatment based on qRT-PCR, i.e. a time period where persister cells are likely still forming and the overall CFU/ml is sufficiently high, indicating that elevated transcription levels of native (i.e. not overexpressed) *hipA* play a role in persister cell formation and thus antibiotic tolerance of *K. pneumoniae* (PMID: 31520807). However, analyzing a homogenous persister cell population, e.g. after antibiotic stress that kills all non-persister cells, is immensely challenging because the number of persister cells is extremely low. In an attempt to address the Reviewer's question, we initiated a preliminary experiment, which is based on a very sensitive split complementation of two nanoluc (luciferase) fragments analogous to the split GFP system. For this, *hipA*$_{kp}$ was fused to the small HiBiT tag on the chromosome allowing us to measure native $HipA_{kp}$ protein levels as *hipA*$_{kp}$ is under control of the native promoter. The HiBiT Lytic Detection System (Promega) was then used, which contains the LgBiT part of the luciferase and substrate. If HipAkp is present in the cell lysate a luminescent signal can be measured, which is proportional to the amount of the protein of interest over a wide range. *K. pneumoniae* wild type was used as a negative control which was CFU-adjusted to the mutant expressing *hipA*$_{kp}$-HiBiT for the measurement. Though the overall luminescent intensity is very low – which is not surprising, as *hipA* is not expressed at high levels in other bacteria – there is an increase in signal intensity and thus *HipA*$_{kp}$ levels in response to ciprofloxacin at 30 and 60 min, when the majority of bacteria have been killed **(Rebuttal letter Figure 2**).

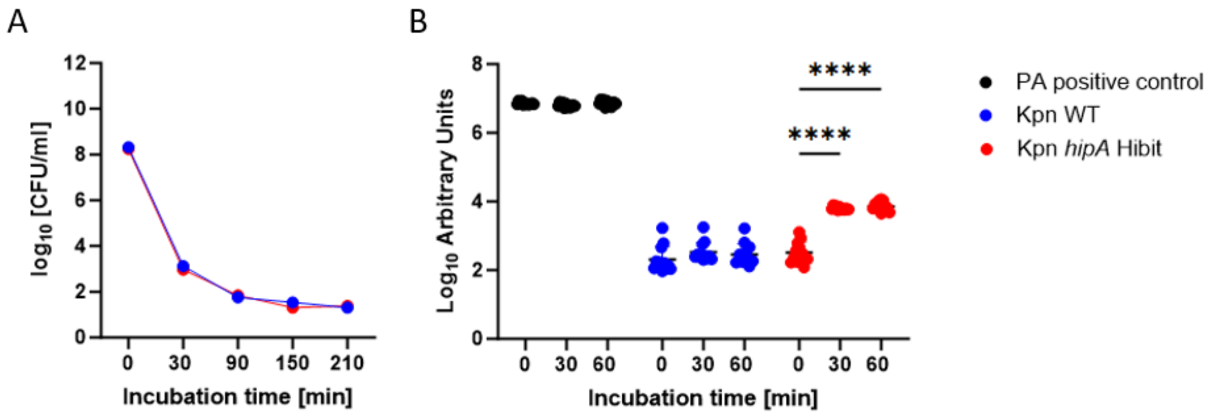


**Rebuttal Letter Figure 2. A.** CFU/ml of the indicated *K. pneumoniae* strains after incubation with 5 μg/ml ciprofloxacin. **B.** Luminescence of the indicated *K. pneumoniae* strains after 0, 30- and 60-min incubation with 5 μg/ml ciprofloxacin and addition of the LgBiT to the lysates. A *P. aeruginosa* mutant constitutively producing a HiBiT-tagged protein (gift by Dr. Erwin Bohn) served as a positive control.

5. The authors speculate (line 344 and the subsequent paragraph) that numerous pathways are potentially post-translationally regulated by HipA. This statement can be easily backed up by normalizing the phosphoproteomics to proteomics – are the increases in phosphorylation of specific sites independent of the increase in protein levels? (moreover, both proteome and phosphoproteome changes could be compared to the transcriptome profiles, but this would of course require additional experiments so may be beyond the scope of the current work).

We thank the Reviewer for the suggestion: we agree that it is important to check and report the phosphorylation levels of specific sites after normalization to protein levels. Since the number of proteins that appear to be regulated upon HipA overexpression is low and it is known that such normalization leads to reduction of data points, we did not normalize the phosphoproteomics data to the proteome in the submitted version of the manuscript. However, as requested by the Reviewer, we re-analyzed the data and now report both the normalized and unnormalized sites in the revised version of the manuscript. As expected, upon normalization we lose numerous phosphorylation sites due to the absence of corresponding protein data. However, the reported upregulation of phosphorylation was independent of protein levels. Therefore, the conclusions of the study did not change and were even corroborated by the normalization. A direct comparison of unnormalized and normalized phosphoproteomics data is shown in the **Rebuttal letter Figure 3 and Supplementary Figures S2E, S3E, S3F, and S4G.** Though this study focuses on post-translational regulation, we agree with the Reviewer that it would be interesting to investigate the proteome and phosphoproteome in the context of global gene expression patterns in future work.

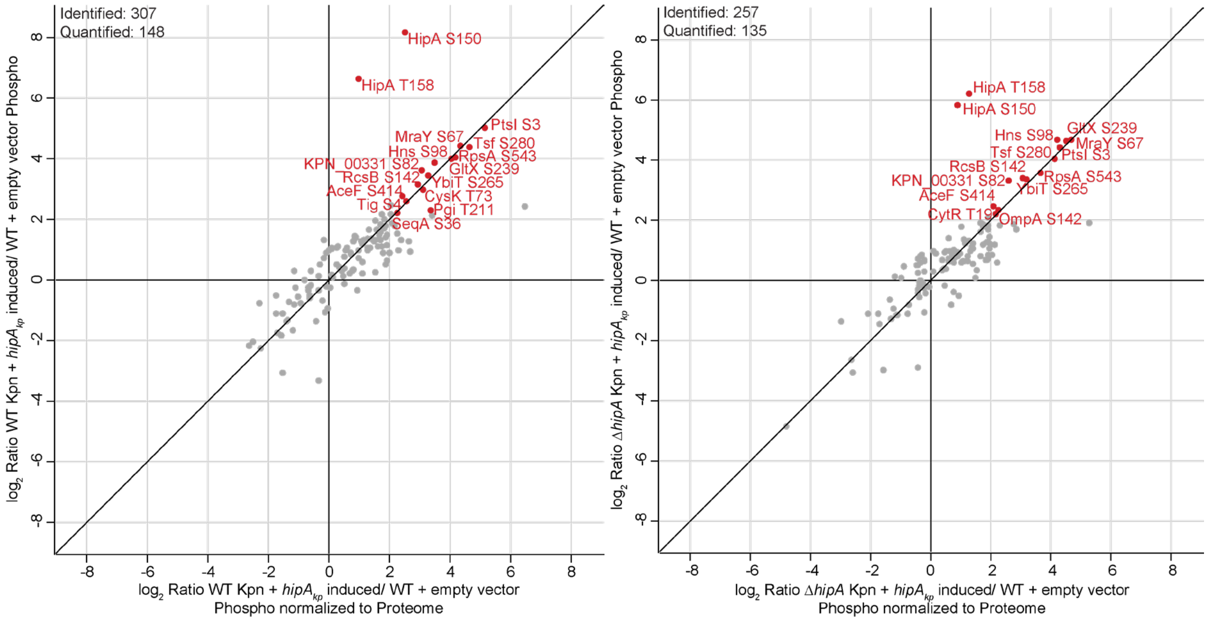


**Rebuttal letter Figure 3.** The ratio of normalized (x-axis) and unnormalized (y-axis) phosphoproteomics data obtained from *K. pneumoniae* carrying empty vector versus overexpressing *hipA*$_{kp}$ in WT and Δ*hipA*.

Reviewer #4: Specific comments

- In the Results, the flow of information is logical and well-presented but the switch from Klebsiella in 1.1 to E. coli 1.2 and then back to Klebsiella in 1.4 is not intuitive. If the E. coli overexpression of hipA is known, should section 1.2 and 1.3 be presented in supplemental or include Klebsiella within these sections? If hipA is well characterized in E. coli and the point of the manuscript is to provide novel information for Klebsiella, this point is diluted with the current approach.

We appreciate the reviewer's suggestion to reorganize the paragraphs for enhanced clarity. We would like to stress that expressing *hipA*$_{kp}$ in *E. coli* is novel and - following the observation of the sequence similarity of HipA$_{ec}$ and HipA$_{kp}$ - was done to provide phenotypic evidence for the functional similarity of HipA in *E. coli* and *K. pneumoniae*. However, we agree with the Reviewer and do see the need to simplify this part of the manuscript. We have now organized the results based on *hipA*$_{kp}$ overexpression effect on: 1) phenotypes and 2) proteome/phosphoproteome in both organisms. This reduced the number of result paragraphs from nine to six. We would like to emphasize that all data presented in the manuscript are novel for *K. pneumoniae*.

- Was the phosphoproteome data normalized to the total proteome? If not, then a consideration to possible elevated overproduction in the total proteome influencing the phosphoproteome levels should be presented. Is this what line 632 refers to?

We thank the Reviewer for raising this point and we kindly refer the Reviewer to Rebuttal Letter Figure 3 and the corresponding reply to the comment made by Reviewer #3. Initially, we did not normalize the phosphoproteome data to the total proteome, as we only observed minor changes in the latter. However, we now follow the suggestions by the Reviewer #3 and Reviewer #4 and report the normalized and

unnormalized phosphoproteome data in the main figures 3 and 4 and directly compare them in supplementary figures S2E, S3E, S3F, and S4G. As expected, normalization led to a fewer number of data points but did not influence reported phosphopeptide ratios.

- Line 549: how many biological replicates were used?

Two to three biological replicates for each experiment were used for cell lysis and protein extraction to perform mass spectrometry analysis as indicated in figure legends and summarized in the Supplementary Table S3. Each replicate was digested with two different enzymes (trypsin and chymotrypsin) to increase the overall coverage of the phosphorylation sites in the phosphoproteome and provide technical replicates. Therefore, each condition was measured 4-6 times.

- There are limitations of identifying phosphosites in Klebsiella using TiO2 beads References: https://pubmed.ncbi.nlm.nih.gov/25156620/; https://pubmed.ncbi.nlm.nih.gov/30231186/; https://pubmed.ncbi.nlm.nih.gov/29377012/). Were the authors able to detect phosphohistidine and phosphoaspartate residues with the acidified samples? Re-searching the total proteome data bioinformatically within these as modifications may provide a new level of insight into the study.

We thank the reviewer for this suggestion. We did not include N-phosphorylated residues in the processing, as we mainly focused on the Ser/Thr HipA kinase and it is widely documented that N-phosphorylation is unstable under pH=8 (the pH in our analysis was significantly lower). Nevertheless, following the Reviewer's comment we did re-process the Experiment 1 with pHis pAsp as an additional variable modification. The results are summarized in **Rebuttal letter Figure 4A**. A total of 593 p-sites were identified, of which 557 p-sites remained after filtering out diagnostic peak, reverse, and contaminants. Out of these, 338 (60%) had a localization probability of > 0.75. Out of 127 pHis and pAsp sites, only 52 had a localization probability above 0.75. The distribution of phosphorylated amino acids can be seen in the **Rebuttal letter Figure 4B**. 85% of the sites displaying a localization probability of > 0.75 belong to Ser, Thr or Tyr, and approximately 15% are His and Asp. Of note, the scores and PEP values of H/D-phosphorylated peptides were very low, pointing to high frequency of false ID or mis-localization of phosphorylated residues. For these reasons, we specifically addressed O-phosphorylated residues S/T/Y in the manuscript.

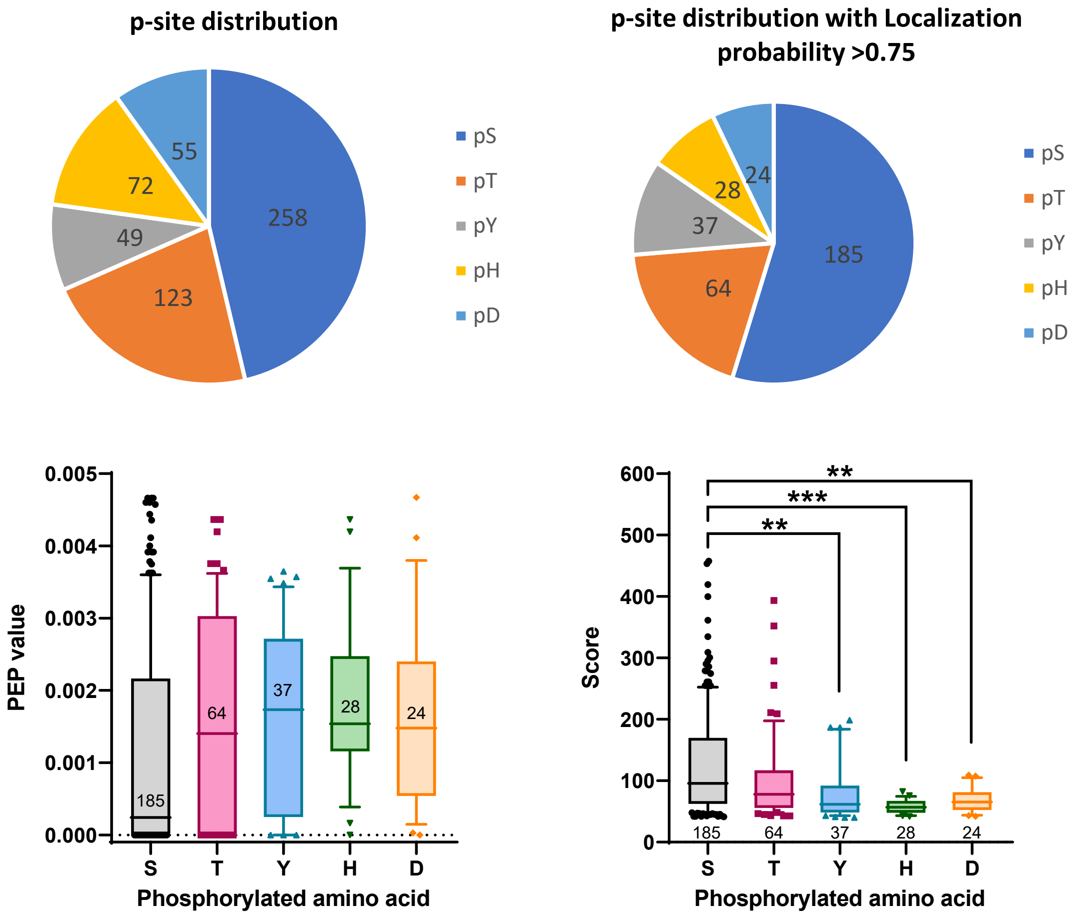


**Rebuttal letter Figure 4. A.** P-site distribution of all identified phosphorylation sites across different amino acids in experiment 2.1 (Left). P-site distribution after filtering out the p-sites with localization probability lower than 0.75 (Right). **B.** Distribution of PEP values (Left) and peptide scores (Right) in different phosphorylated amino acids.

- For robustness in data analysis, significantly different sites should be determined following a statistical test (e.g., Student's t-test, Welch's t-test) and multiple hypothesis testing correction with a false discovery rate at 5% or less. From line 644, was this correction performed? If not, it should be and data should be presented as those that reach the FDR and those that do not.

We thank the reviewer for raising this point. We now include the Student's One sample t-test in Suppl. Fig. S2, S3 and S4 and plot the negative $Log_{10}$ of the Benjamini-Hochberg corrected (FDR<0.05) t-test p-value against the $Log_2$ of the t-test difference (corresponding to $log_2$ fold change). As expected, strict requirement for a multiple hypothesis testing correction led to a loss of important information due to missing values in the phosphoproteome datasets, which are intrinsically noisy and have fewer data points then proteome datasets. This can be seen from phosphorylation site S239 on GltX, which appears not to be significantly regulated despite its high fold change and the fact that it is a known HipA substrate (this phosphopeptide did not have a ratio in 2/3 replicates due to missing intensity in "L" channel) **(Rebuttal**

**letter Figure 5)**. Due to this fact, we decided to show BH-corrected volcano plots for the proteome data and scatter plots for the phosphoproteome data.

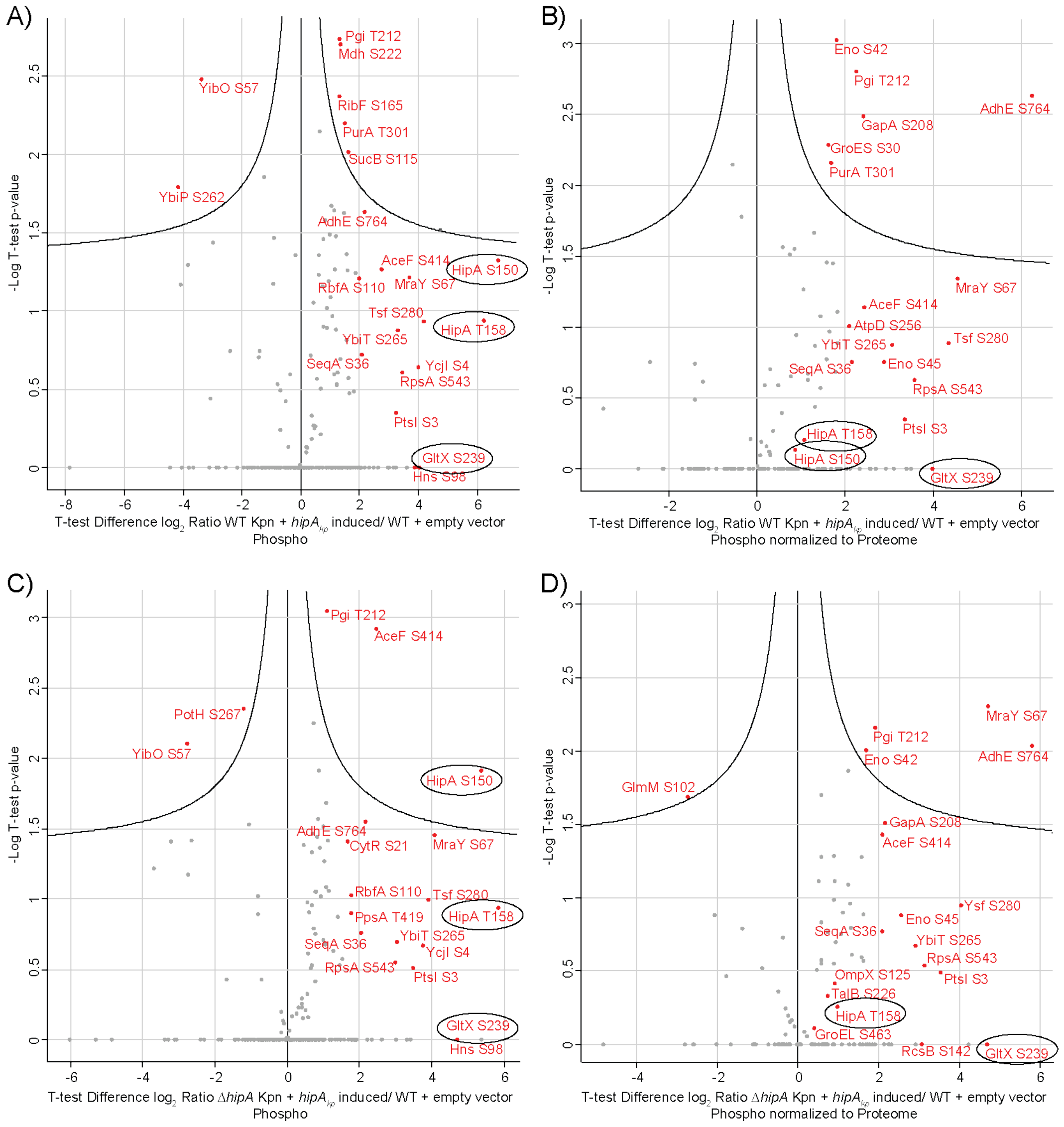


**Rebuttal letter Figure 5.** Volcano plots showing differential phosphorylation site expression, with the x-axis representing the T-test difference in $\log_2$ ratios of WT (A and B) and Δ*hipA* (C and D) *Kpn* overexpressing *hipA$_{kp}$* versus empty vector, and the y-axis displaying the negative log10 of the Benjamini-Hochberg corrected t-test p-value (p = 0.05). Plots A and C show unnormalized phosphoproteome data, while plots B and D are normalized to the proteome.

- For robustness in data analysis, localization probability of >75% is needed for accurate phosphopeptides identifications. Was this performed? It is not clear from the methodology.

In the submitted version of the manuscript, the number of p-sites with a localization probability of > 0.75 for each experiment is indicated in Supplementary Table S4. It showed that around 70% of the total p-sites identified have a localization probability >0.75. However, for simplicity, we have now filtered the

phospho data for localization probability >0.75 and only show these phosphorylation sites in the scatter plots in the revised manuscript (Figure 3C-3F, 4D and 4E).

- Fig. 5f – great to see the comparison to other studies; however, this figure is misleading as the same statistical testing and robustness was not presented in the current study. This needs to be completed for an accurate comparison.

For this comparison we considered all phosphorylation sites reported by different studies before any statistical testing or filtering for localization probability. We note that it is almost impossible to perform a "fair" comparison, as the two studies by Li *et al.* used a different strain of *K. pneumoniae* and different software for data processing and analysis, which might have resulted in some differences in protein annotation and position of phosphorylation sites. However, despite these and probably other differences between the previously published studies and our study (growth conditions, etc.) we think it is important and insightful to compare all the data.

**Part III – Minor Issues: Editorial and Data Presentation Modifications**
Please use this section for editorial suggestions as well as relatively minor modifications of existing data that would enhance clarity.

Reviewer #1: (1) I do not find the figure legends.

In the revised version of the manuscript, the figure legends and figures can be found in the text; in addition, figures are uploaded as separate files.

(2) Line 636, "Re-quantify" should be modified to the noun form.

By "Re-quantify" we meant that the MaxQuant option "Re-quantify" was ticked during processing.

Reviewer #2: None

Reviewer #3: (No Response)

Reviewer #4: - Add italics and proper formatting to scientific names within the references.

We thank the Reviewer for pointing this out. We have now corrected the formatting.

- Fig. 2A – plots are not clear as to how they represent growth curves. Suggest revising. Connection to 2B is unclear. Same for Fig. 3A. What value do these plots add? Fig. 3B is a clear growth curve.

We thank the Reviewer for pointing out that the term "growth curve" is not appropriate for some of the graphs, since the growth was measured over a short period of time. We have now changed "growth curve" to "growth" where necessary to correct for this. The connection between Figure 2A and 2B is that they show data of the same culture and conditions as indicated in the graph legend but measure different outcomes. In Figure 2A the $OD_{600}$ was measured to analyze growth. However, by measuring OD it is not possible to differentiate between viable and intact dead bacteria. Quantifying specifically viable bacteria, the number of CFU was determined and shown in Figure 2A with Viability heading. Thus, each graph answers a different question relevant to the conclusions of this study. The same holds true for the graphs shown in Figure 2C (former 3A). Figure 2D (former 3B) shows a growth curve to answer the question

whether $HipB_{kp}$ the putative antitoxin can counteract the toxicity of $HipA_{kp}$, which is different from the question if overproduced $Hip_{kp}$ has a toxic effect in the first place answered in Figure 3A. To make this clearer we have now added "Complementation assay" as the title for Figure 3B. We also revised the x-axis of plots in Figures 2, 4A and 4B to a time scale.

- Fig. 2C and D, 3C and D, and 4C and D – why not present as volcano plots with complete statistical power?

For the proteome data, we have plotted the volcano plots, which are shown in Figures 3A, 3B and 4C in the manuscript. However, for phosphoproteome data many proteins have no ratios in different replicates due to missing values for intensity in the Light (control) channel (where the HipA kinase was not overproduced). Therefore, we would lose important information if we plot volcano plots. In addition, the known HipA substrates (positive controls) do not appear above significance threshold, likely due to fewer data points and intrinsically noisy data in phosphoproteome measurements. Therefore, we hope the Reviewer agrees that it is better to show scatter plots for intensity vs. ratio using the average median values from multiple replicates for manuscript Fig 3C, 3D and 4D, as has been done in previous studies (PMID: 30206139, 36537800). Phospho-sites above $\log_2$ value 2 (four-fold) are marked in the manuscript to be up-regulated upon overexpression compared to empty vector. Moreover, we showed multi-scatter plots to demonstrate the good correlation between the replicates (Fig S3B) and the overlap between the phospho-sites obtained from different experiments with hipA overexpression (Fig 5A, 5B).

- Growth curves for Fig. 4?

It is possible that the Reviewer is suggesting to extend the incubation times under antibiotic treatment beyond the indicated time intervals. However, because of the strong growth differences between the strains and conditions using the indicated incubation times we did not deem necessary to do this. It is also possible that the Reviewer is referring to growth analysis of *K. pneumoniae* when exposed to gentamicin and ciprofloxacin. OD values of *K. pneumoniae* cultures after treatment with varying concentrations of the antibiotics are shown in the supplemental material. We have changed the headings of the growth curves to OD and CFU/mL to make this clearer.

- The figure legends are within the main text but without the figure itself – suggest revising.

We have now moved the main figures along with figure legends in the main text. We have also uploaded each figure separately.

- Supp. Fig. 2 – are these representative of volcano plots? Why present in this manner?

These are scatter plots based on ratios between two conditions to better visualize regulated proteins. Many previous studies have been published with phosphoproteome data presented in scatter plots (see our comment above). To make data more complete, we have now plotted the average median values from multiple replicates and also included phospho normalized to the proteome plot.

- The PRIDE data set is not publicly available and therefore, the login information and password for reviewers is needed.

We provided the login information to the Editor. To ensure that all the Reviewers can access the data sets, we provide the login details: (Username: reviewer_pxd051521@ebi.ac.uk and Password: UF5cpxTa)
